# Innate Immune Sensors and Gastrointestinal Bacterial Infections

**DOI:** 10.1155/2011/579650

**Published:** 2011-05-19

**Authors:** Georgina L. Hold, Indrani Mukhopadhya, Tom P. Monie

**Affiliations:** ^1^Division of Applied Medicine, University of Aberdeen, Aberdeen AB25 2ZD, UK; ^2^Department of Biochemistry, University of Cambridge, 80 Tennis Court Road, Cambridge CB2 1GA, UK

## Abstract

The gastrointestinal microbiota is a major source of immune stimulation. The interaction between host pattern-recognition receptors and conserved microbial ligands profoundly influences infection dynamics. Identifying and understanding the nature of these interactions is a key step towards obtaining a clearer picture of microbial pathogenesis. These interactions underpin a complex interplay between microbe and host that has far reaching consequences for both. Here, we review the role of pattern recognition receptors in three prototype diseases affecting the stomach, the small intestine, and large intestine, respectively (*Helicobacter pylori* infection, *Salmonella* infection, and inflammatory bowel disease). Specifically, we review the nature and impact of pathogen:receptor interactions, their impact upon pathogenesis, and address the relevance of pattern recognition receptors in the development of therapies for gastrointestinal diseases.

## 1. Introduction

Microbes play key roles in both health and disease. This is particularly relevant in the gastrointestinal (GI) tract, where the high bacterial load (up to 10^12^ bacteria/ml) ensures a continual source of immune stimulation. The gastrointestinal immune system is intricately designed not only to identify and isolate pathogenic organisms but also to continue a predominantly symbiotic host-bacterial relationship that often exists for mutual benefit. The outermost sentinels are the intestinal epithelial cells (IECs) that line the gut and act as a physical barrier between the luminal contents and the host immune system. However, these cells are not passive players and contribute to the innate immune response by expressing pattern recognition receptors (PRRs) that can identify and respond to microbial organisms. They also express major histocompatibility antigens and act as antigen-presenting cells bridging innate and adaptive immunity. Further specialised antigen presenting cells include M cells in the epithelial lining and dendritic cells in the lamina propria. Together, these contribute key innate and adaptive immune responses [1, 2].

T cells are the major cellular components of the adaptive response and are located between epithelial cells (intraepithelial lymphocytes) and in the lamina propria. Nomadic intraepithelial lymphocytes contribute barrier functions and help maintain intestinal epithelium integrity. Several T cell subsets (T_H_1, T_H_2, and T_H_17) are present in the lamina propria resulting in distinct and characteristic cytokine responses that help prevent systemic spread of pathogens. Activation of B cells, often within Peyer's patches in the lamina propria resulting in antibody production and secretory IgA, for example, has a key role in influencing luminal events. The commensal gut microbiota paradoxically influence maturity and the development of this local immune system [3]. The host in turn develops “tolerance” to this large and diverse group of bacteria. This relative unresponsiveness is mediated by another group of cells called T regulatory cells (Tregs) [4]. The current paradigm of immune tolerance suggests that commensal bacteria may in fact influence and promote differentiation of Tregs resulting in a mechanism to tolerate foreign antigens of the microbiota [5]. These areas of research are likely to be crucial in enabling us to comprehend the molecular mechanisms by which the gut microbiota conducts and regulates immune homeostasis.

As outlined above the interaction of commensal and pathogenic bacteria with PRRs influences both the immediate and adaptive immune responses. This can affect the nature of the commensal species and the outcome of pathogenic infection. In this paper, we will focus on the contribution of PRRs in diseases of three anatomically diverse sites within the GI tract. Specifically, *Helicobacter pylori* and the stomach*, Salmonella spp.* and the small intestine, and inflammatory bowel disease and colonic microbiota. We will discuss the contribution of pathogen:PRR interaction to pathogenesis and consider how understanding PRR:pathogen interplay can aid therapeutic treatment and development. 

PRRs consist of four main receptor families: Toll-like receptors (TLRs), nucleotide-binding domain leucine-rich repeat containing receptors (NLRs), RIG-I like receptors (RLRs), and the C-type lectin family [6]. The membrane bound TLR and cytosolic NLR family members are particularly relevant for the three diseases under discussion. Various molecules act as PRR stimuli in each of these diseases (Table 1) [6, 7]. Whilst PRRs are known to be found in immune cells such as macrophages and dendritic cells, their presence on other cell types, including gastrointestinal epithelial cells, which are likely to come in to contact with microorganisms, is less well understood.

Expression of TLRs is nonuniform throughout the gastrointestinal tract with expression of TLRs in the oesophagus having not been documented to date. In the stomach, expression of TLR2, 4, 5, and 9 has been demonstrated [8–10] (Table 2). TLR expression in the intestines is more extensive although under normal physiological conditions, TLR expression in IECs is downregulated [11]. 

PRRs respond to evolutionarily conserved ligands whether derived from friend or foe. The host must retain the ability to detect and mount an appropriate immune response to pathogenic bacteria, whilst simultaneously avoiding inappropriate, or excessive, responses to the commensal microbiota [12]. Many mechanisms are employed within the GI tract to facilitate successful discrimination. These include PRR downregulation from the apical epithelial surface, PRR signaling crosstalk, requirements for multiple PRR activation, and negative regulation of PRR signaling (reviewed in [13]). Understanding the role of the PRRs of the innate immune system in the homeostatic and pathogenic functioning of the GI system is essential if we are to successfully manage and treat diseases that already place a significant burden on the healthcare system.

## 2. PRRs and *Helicobacter pylori*

**Figure 1:**
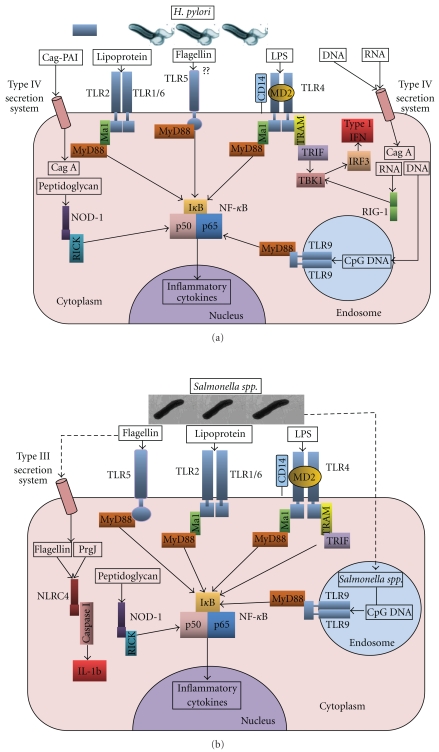
The interaction of *Helicobacter pylori *and *Salmonella spp. *with pattern recognition receptors. Schematic representations of the PRRs, activating ligands, and simplified activation pathways for innate immune signaling for (a) *Helicobacter pylori *and (b) *Salmonella enterica serovar Typhimurium*. Both pathogens activate multiple PRRs found on cellular membranes and in the cytoplasm. Together, this instigates a complex interplay of signaling crosstalk that influences the host response to the pathogen and results in induction of a proinflammatory immune response. In the case of *Salmonella spp.,* activation of caspase-1 via NLRC4 ultimately leads to cell death via pyroptosis. *H. pylori *electron micrograph image provided courtesy of Professor Dave Kelly, University of Sheffield, UK. Electron micrograph image of *Salmonella enterica serovar Typhimurium *provided courtesy of Professor Sangwei Lu, UC Berkeley.

*Helicobacter pylori* is a ubiquitous, noninvasive Gram-negative bacterium that colonises the stomach of nearly half of the world's population [14]. It is associated with chronic gastritis, duodenal ulcer, gastric ulcer, gastric adenocarcinoma, and gastric MALT lymphoma*. H. pylori* organisms reside in the gastric acidic milieu, just below the mucous layer in close proximity to the gastric epithelium. Infection outcome is determined by both bacterial pathogenicity factors and host susceptibility factors [15, 16]. Innate immune receptors play an integral role in recognition and subsequent pathogenesis of *H. pylori*. Infection is established following adherence of the bacteria to the gastric mucosal cells. *H. pylori* has a tropism for gastric mucosal cells suggesting the presence of receptors that aid in docking on to the epithelial surface. (Table 1 and Figure 1(a)). Of the cell surface PRRs, TLR2, 4, and 5 have been studied most extensively in the context of *H. pylori *infection. Initial studies by Backhed et al. showed that TLR2, TLR4, and TLR5 were detected in biopsies taken from patients with *H. pylori* infection, but TLR4 was not found in the subset of antral gastric epithelial cell preparations [17]. The investigators then challenged TLR4 expressing cells with both *cag*PAI positive and negative *H. pylori*. The former strains elicited an IL-8 response, whereas the latter did not, suggesting that response to *H. pylori* was *cag*PAI dependant and independent of TLR4. In contrast, a similar study utilising clinical isolates of *H. pylori* showed that cytokine-inducing activity of *H. pylori* LPS was mediated by TLR4 [18]. Utilising TLR4^−/−^ macrophages, these investigators further demonstrated a lack of response to *H. pylori* lipopolysaccharide (LPS), confirming the importance of TLR4. Surprisingly, the cytokine response to whole *H. pylori* bacteria was mediated not by TLR4 but rather by TLR2 [18]. However, subsequent work demonstrated apical and basolateral expression of TLR4 on gastric cells in non-inflamed gastric epithelium and also in patients with *H. pylori* infection [8, 10]. Confocal microscopy also showed direct attachment of *H. pylori* bacteria to the TLR4 receptor at the apical pole [10]. 

As with all Gram-negative bacteria, LPS is a major cell surface component of *H. pylori. *However, *H. pylori *(along with a collection of other Gram-negative bacteria, for example, *Y. pestis*, *P. gingivalis*) have evolved mechanisms to modify the structure of their LPS (in particular the lipid A component) in different environments [20]. Different lipid A structures have different binding affinities to TLR4 complex constituents, and this can lead to altered host recognition. For example, lipid A of *Helicobacter pylori* in comparison to that of *Salmonella spp.*, contains a major monophosphorylated tetra-acylated lipid A species which is not thought to bind efficiently to the TLR4/MD-2 receptor complex due to the lack of a phosphate group in the lipid A anchor [21]. This loss of one or both phosphate groups from the lipid A anchor is seen in a number of pathogenic organisms. *H. pylori* tetra-acylated LPS has poor activity against TLR4. However, clinical *H. pylori *isolates have been shown to possess the less abundant hexa-acylated form and consequently are much more effective at activating the TLR4/MD-2 receptor complex. Differential LPS expression seen between different *H. pylori *strains could explain the conflicting evidence regarding recognition of *H. pylori* LPS by TLR4. 

TLR5 and TLR9 have also been identified in non-inflamed gastric epithelium and in *H. pylori*-associated chronic gastritis. The localization of TLR5 and TLR9 was found to be exclusively basolateral in *H. pylori*-associated chronic gastritis [10], suggesting that these two TLRs may not be essential for *H. pylori* recognition [2]. This was further confirmed by demonstration that aflagellated *H. pylori* mutants still induced IL-8 production [22]. TLR5 does interact with *H. pylori* flagellin, but the interaction induces only weak receptor activation [23]. This was confirmed by an elegant study in which TLR5 responsive motifs in *Salmonella* flagellin were replaced with the equivalent regions from *H. pylori*. This rendered the flagellin inactive against TLR5 [24]. Evasion of TLR5-mediated bacterial detection may be a major mechanism for immune escape and persistence by *H. pylori*. 

The involvement of TLR9 in *Helicobacter *pathogenesis has not been extensively studied. A recent murine study, by Rad et al. [25], identified the potential of* H. pylori *DNA to act as a TLR9 ligand. This work also reported the induction of proinflammatory cytokines and type I IFN-stimulated genes in murine dendritic cells by *H. pylori *RNA in a TLR- and RIG-I-dependent mechanism, respectively. This study utilised direct transfection of *H. pylori* nucleic acid to activate the relevant receptors. Whilst dendritic cells appear capable of efficiently internalising live *H. pylori*, the precise mechanisms by which ligands are presented to intracellular PRRs remain to be clarified.

The ability of other immune cell types to respond to *H. pylori *has also been documented. Natural killer (NK) cells respond to *H. pylori*-specific membrane-bound protein HpaA to produce interferon gamma (IFN*γ*) following activation of either TLR2:TLR1 or TLR2:TLR6 heterodimers [26]. The exact relevance of NK cell activation by *H. pylori* in infection is not known. However, NK cells may gain access to the *H. pylori* antigens in the mucosa as a result of leaky intercellular tight junctions. Given that gastric cancer is one of the known sequelae of *H. pylori* chronic gastritis one can speculate that *H. pylori*-mediated NK cell activation and subsequent IFN*γ* production could contribute to tumour immunosurveillance.

The ability of *H. pylori* to activate intracellular PRRs also extends to the cytoplasmic NLR family. Current evidence suggests that cytoplasmic PRRs are exposed to *H. pylori* either following endocytosis or via the action of the *cag*PAI encoded type IV secretion system (Figure 1(a)). This system allows bacterial proteins such as CagA as well as peptidoglycan fragments to be injected into the epithelial cell cytosol. An alternative method for peptidoglycan entry into cells has recently been proposed by Kaparakis et al. [27]. They suggest that bacterial outer membrane vesicles, containing peptidoglycan fragments, associate and fuse with lipid rafts on the host cells and divest their contents into the cytoplasm. It may well be that both these mechanisms are employed during infection. Once in the cell, *H. pylori *peptidoglycan is recognised by the NLR protein NOD1 [28]. Activation of NOD1 leads to receptor oligomerisation and the recruitment of the cellular kinase receptor interacting protein 2 (RIP2). RIP2 is activated via induced proximity and subsequently targets IKK (inhibitor of nuclear factor kappa B (NF-*κ*B) kinase) *γ* for degradation, allowing IKK*α* and IKK*β* to phosphorylate I*κ*B and initiate NF-*κ*B release and nuclear translocation. The use of the adaptor CARD (caspase activation and recruitment domain) 9, rather than RIP2, activates the stress kinase pathway. Both pathways initiate a strong proinflammatory immune response which includes the production of antimicrobial *β*-defensins, thereby helping to mediate bacterial killing [29, 30]. NOD2 signalling follows a very similar pathway to NOD1.

The presence of functional polymorphisms in genes of the innate immune system can affect pathogenesis and the magnitude and direction of the host's response to infection. They may also impact on the effectiveness of subsequent therapeutic strategies [31]. Hundreds of single nucleotide polymorphisms (SNPs) have been identified in PRRs, but the functional consequences of only a few have been defined. The TLR4 polymorphism (TLR4 Asp299Gly) decreases responsiveness to LPS [32] and has a positive correlation with susceptibility to several infectious diseases including *H. pylori*-induced gastric cancer [33]. Other functional PRR polymorphisms are also associated with *H. pylori-*induced disease: the −1237T/C promoter polymorphism in TLR9, the −159C/T promoter polymorphism in CD14 (a component of the TLR4 receptor complex), the TLR2 −196 to 174 deletion promoter polymorphism, and the Glu266Lys NOD1 polymorphism have all been associated with increased risk of *H. pylori *attributable disease although this risk is not always present at all disease stages [34–37]. 

In conclusion, it is quite evident that the innate immune system and its wide array of PRRs play a significant role in recognising *H. pylori* and triggering antimicrobial host defense responses. Unlike other Gram-negative bacilli such as *Salmonella spp.*, the immune response against *H. pylori* LPS and flagellin is not as robust and is less defined. This may well be a result of immune escape mechanisms employed by the bacteria to aid persistence. Better understanding of these pathogenic mechanisms will help in targeting PRRs to develop new therapeutic strategies against *H. pylori* and other bacterial pathogens.

## 3. The Role of PRRs in *Salmonella spp.* Pathogenesis

*Salmonella spp.* are versatile enteric pathogens that employ multiple virulence determinants to survive within the host and contribute to pathogenesis. These include *Salmonella* pathogenicity island (SPI)-1 and SPI-2-encoded type III secretion systems (T3SS) and bacterial factors such as LPS and flagellin. Interaction with the host is a critical element in *S. Typhimurium* pathogenesis influencing progression and outcome of infection. Uptake into epithelial cells early in infection, or subsequent entry into macrophages, contributes to PRR stimulation and plays an important role in balancing host:pathogen interplay. The key *Salmonella* ligands and responsive PRRs are outlined in Table 1 and Figure 1(b). 

TLR2 and TLR9 both contribute to detection of *Salmonella* and control of pathogenesis. TLR2 contributes more significantly at high, rather than low, bacterial loads [38, 39]. TLR9, in turn, is activated by CpG-rich repetitive DNA elements in the bacterial genome and initiates a strong interferon-*α* response [40]. The major TLRs involved in interaction with *Salmonella* are, however, TLR4 and TLR5. These respond to LPS and flagellin, respectively. Mice defective in TLR4 show an impaired response to *S. Typhimurium *infection [39]. Purified LPS from *Salmonella *induces high levels of tumour necrosis factor (TNF)-*α* and nitric oxide in a manner very similar to direct infection of macrophages. Activated macrophages also upregulate mitogen-associated protein kinase JNK and p38 though this appears to be TLR4 independent [41]. The use of TLR adaptor proteins is influenced by the bacterial load of *Salmonella* infection. For instance, the control of bacterial growth during murine infection is critically regulated by TLR4 and MyD88 (myeloid differentiation factor 88), and not Mal (MyD88 adaptor like protein) or TRIF (Toll interleukin-1 receptor containing adaptor inducing IFN-*β*). TRIF is, however, important for bacterial killing very early during infection [38, 39]. Mal is also essential for a full interleukin (IL)-6 response at low, but not high multiplicity of infection [42]. The variation in activation threshold and sensitivity for the different TLRs to *Salmonella spp.* presumably helps regulate the host response, whilst maintaining the ability to respond rapidly to the high threat associated with a large bacterial load. One could postulate that a similar mechanism may contribute to the discrimination between commensal and pathogenic bacteria and the maintenance of homeostasis. What is certainly true is that the cellular response to the detection of *Salmonella* plays an important role in the progression and manifestation of immunopathology.

*Salmonella *flagellin can interact with TLR5 on the basolateral surface of intestinal cells and also on dendritic cells in the lamina propria. This interaction involves conserved regions of FliC [24]. This activation could conceivably involve direct bacterial contact; however, *Salmonella spp.* actively secrete components of the flagella, such as FliC, via the SPI1 T3SS (Figure 1(b)). These components can activate TLR5 independently of direct bacterial contact. The importance of this interaction has been highlighted through studies with aflagellated bacteria investigating *Salmonella*-mediated gastroenteritis and typhoid fever [43, 44]. Loss of flagella correlated with a reduction in the initial inflammatory response to infection followed by a subsequent increase in bacterial load, enhanced inflammation, and greater disease severity. It would therefore seem that, just like TLR4, TLR5 is a key mediator in the interactions between host and *Salmonella spp. *and is vital in regulating disease burden and bacterial replication.

Flagellin subunits are also delivered directly to the cytoplasm by the SPI1 T3SS, where they activate the intracellular receptor NLRC4 (also known as IPAF) in a TLR5-independent manner [45, 46]. This results in the formation of an inflammasome and the recruitment and processing of procaspase-1. Active caspase-1, subsequently, cleaves pro-interleukin 1*β* and 18 into active proinflammatory cytokines, which are secreted from the cell. Caspase-1 activation can also lead to cell death via pyroptosis [47]. NLRC4 can also be directly activated by the T3SS component PrgJ [48]. The interaction between *Salmonella spp. *and NLRC4 is complex. Expression of bacterial stimuli is temporal and, consequently, affects the likelihood of detection and immune clearance. Attenuation of flagellin and PrgJ expression could be viewed as an immune evasion strategy comparable with LPS variation in *H. pylori*. It is conceivable that NLRC4 activation contributes to bacterial pathogenesis as canines, which appear to be inherently resistant to *Salmonella*-mediated pathology, do not possess a fully functional NLRC4 protein. 

In addition to the NLRC4 inflammasome, *Salmonella *also activates caspase-1 via the NLRP3 inflammasome [49]. Furthermore, peptidoglycan from *Salmonella spp.* is recognized by NOD1 [28, 50]. NOD1 activation has been shown to be important in dendritic cells in the lamina propria [50]. It is clear that the dynamics of PRR:*Salmonella* interaction are complex and contribute to the establishment, progression, and clearance, or otherwise, of infection. As with *H. pylori*, host genetics can influence the progression of Salmonella mediated disease. As yet, there are no published connections between PRR SNPs and disease outcome. However, recently a genome-wide screen linked SNPs in the immune regulator CARD8 with susceptibility to *Salmonella-*mediated cell death [51].

## 4. Pathogenesis of Inflammatory Bowel Disease: Interaction of PRRs with Gut Microbiota

Inflammatory bowel disease (IBD) comprising of ulcerative colitis (UC) and Crohn's disease (CD) is an idiopathic disease characterized by chronic inflammation of the gut. Over the years, several pathogenic theories have been postulated, ranging from psychosomatic to autoimmunity to allergic to genetic predisposition. Our understanding has evolved, and the current consensus of the aetiopathogenesis of these conditions involves one of four components: global changes in the environment, effect of genetic variations, aberrations of innate and adaptive immune responses in handling microbiota, and changes in the luminal microbiota [52]. 

**Figure 2:**
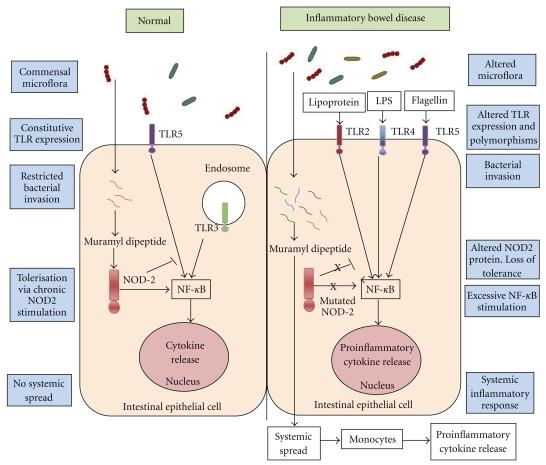
The interaction of colonic microbiota and pattern recognition receptors in normal colonic epithelium and in the epithelium of inflammatory bowel disease. The schematic representation summarizes the key abnormalities at the level of the intestinal lumen, surface PRRs, and intracellular PRRs in inflammatory bowel disease and compares and contrasts them with normal homeostatic interactions.

The colonic epithelium lies in close proximity to a diverse luminal microbiota. This microbial ecosystem is critical in the maintenance of normal homeostasis through their symbiotic contribution to diverse processes that include digestion, absorption, the supply of essential nutrients, and protection from pathogenic microorganisms [53]. In patients with IBD, this delicate balance is disturbed as a result of host immune defects in recognition or impaired clearance of pathogenic microbes [54]. PRRs are essential in distinguishing “friend from foe” in this very complex interaction and hold the key to understanding how genetic factors lead to an abnormal immune environment, wherein normal commensal organisms can lead to pathological chronic inflammation (Figure 2).

The normal colonic epithelium constitutively expresses TLR3 and TLR5, whereas TLR2 and TLR4 are barely detectable [55]. Distinct changes in TLR expression have been documented in IBD. TLR4 is found to be upregulated in both UC and CD, whereas the levels of TLR2 and TLR5 remain unchanged [56]. In IBD, increased TLR2 and TLR4 expression has been documented in the resident macrophages of the lamina propria [57]. These changes can explain some of the abnormal response to the resident gut microbiota. However, it is difficult to elucidate whether the change in TLR expression initiates disease or is an epiphenomenon resulting from proinflammatory cytokine release. For example, both TNF*α* and IFN*γ* have been shown to increase expression of TLR4 and MD2 and hence responsiveness to LPS [58].

The key role of TLR in the pathogenesis of IBD has been elegantly demonstrated in murine models of colitis. TLR4 and MyD88 knockout mice had distinctly less inflammation after chemical induction of colitis with dextran-sodium sulphate (DSS) as opposed to wild-type mice but grew Gram-negative bacilli more frequently from their mesenteric nodes [59]. This highlights the critical role of TLR4 as a first line of defense against potential bacterial pathogens. Impairment of TLR4 function permits bacterial invasion and persistence and could lead to the characteristic inflammation of IBD. The importance of TLR5 in intestinal homeostasis has been effectively demonstrated using microbiota transfer from knockout mice [60]. In this work, loss of TLR5 was associated with development of metabolic syndrome. Interestingly, TLR5 activation by flagellin has been shown not to cause inflammation in normal animals but to enhance the changes associated with DSS colitis [61].

One can postulate that multiple “hits” by different bacterial species may exacerbate and perpetuate the inflammatory cascade through the mediation of TLRs. Intriguingly, the administration of CpG oligodeoxynucleotides, the ligand for TLR9, did not exacerbate inflammation but instead dampened the inflammation associated with experimental colitis [62]. The activation of an inflammatory receptor to dampen the immune response seems counterintuitive. It does, however, highlight the complex interplay between PRR initiated signaling cascades and reinforces the need for further research to improve our understanding of how these receptors function in clinical settings. After all, modulation of PRRs, such as the activation of TLR9, could be the basis for probiotic use in the treatment of IBD. On the other hand, the bacterial composition has also been found to distinctly change in models of experimental colitis, which in itself may alter pathogenesis and the response to therapy. Either TLR2, TLR4, and TLR5 ligands or the PAMP profile was distinctly different in faecal and intestinal biopsy samples from rats with DSS colitis as opposed to controls [63]. It remains to be seen whether this “dysbiosis” is the initiating or a secondary phenomenon in the pathogenesis of IBD (Figure 2). 

The evidence that TLRs play a role in IBD has also been extrapolated from the study of genetic polymorphisms in human subjects. Two common functional polymorphisms of TLR4 (TLR4 Asp299Gly and Thr399Ile) have been associated with both UC and CD in Caucasian populations [64–66]. Polymorphisms in TLR1 and TLR2 genes (TLR1 R80T and TLR2 R753G) have been associated with pancolitis in UC patients [67]. The association of CD with the TLR9 promoter polymorphism (TLR9-1237) discussed previously due to its significance in the context of *H. pylori *infection, which is associated with increased NF-*κ*B-binding affinity, further confirms the role of bacterial DNA sensing in the pathogenesis of IBD [36, 68].

The strongest genetic associations for IBD have, however, been reported for the NLR family member NOD2. Three polymorphisms have been definitively associated with increased susceptibility to CD: Arg702Trp, Gly908Arg, and leu1007fsinsC (a frame-shift mutation that truncates the carboxy terminal 33 amino acids) [69–71]. Polymorphic NOD2 proteins are unable to respond to bacterial muramyl dipeptide (MDP) leading to ineffective downstream signaling of NF-*κ*B [72]. This allows infection and translocation of enteric bacteria to the lamina propria. NOD2 polymorphisms also result in alterations in cytokine expression following exposure of peripheral blood mononuclear cells to MDP [73, 74]. This may explain some of the alteration in cytokine profile typically seen in CD. A decrease in the protective, anti-inflammatory Th-2 cytokine IL-10 has been documented in NOD2 mutants further adding to our understanding of the functional abnormalities characteristic of CD [75]. Counterintuitively, given the potential involvement of dysfunctional NOD2 SNPs, CD is associated with increased NF-*κ*B signaling. Chronic stimulation of NOD2 may, however, act to tolerise cells against bacterial stimulation [76]. Hence, in CD patients with dysfunctional NOD2 this restraint is removed and the inflammatory response from other PRRs increases. Additionally, NOD2 has been implicated in regulation of the cellular process autophagy through potential interaction with ATG16L1 (autophagy-related protein 16-l isoform 1) [77, 78]. ATG16L1 has also been identified as a CD susceptibility gene [79]. The NOD2 CD-associated SNPs appear defective in the initiation of autophagy, which also functions as an antibacterial defense, and it may be that this is another facet to the role of NOD2 in the pathophysiology of CD. 

Despite the fact that nearly 100 independent genetic loci have been identified as potential risk factors for the development of IBD, there are still a large proportion of cases where these defects have not been identified [80]. These could represent lacunae in our current knowledge but could also underlie the importance of the resident gut microbiome that may well be the “environmental factor” that influences and directs the inflammatory response. PRRs form an intrinsically critical factor in the interface between luminal microbiota and the adaptive immune system and can be useful targets in ameliorating the chronic aberrant immune response in IBD.

## 5. Therapeutic Implications of PRRs for Gastrointestinal Disease

The intricate involvement of PRRs in immune inflammatory pathways makes PRRs attractive targets for the development of therapeutic strategies. To date, most efforts have centered on TLRs. Therapeutic approaches to GI tract diseases are inherently complex. The vast bacterial diversity of resident gut microbiota and the continual exposure to high and variable concentrations of PRR ligands results in continual stimulation from commensal and pathogenic organisms. Consequently, it is difficult to design therapeutic interventions that would specifically target pathogenic PRR responses. One must also consider how the resident microbiota may respond to therapies that could potentially disrupt its homeostatic balance.

TLR therapeutic approaches under development include the use of both agonists and antagonists. For example, TLR9 agonists are undergoing Phase I/II trials for colorectal cancer with the hope of optimising drug-induced inflammatory responses for optimal clinical benefit [81]. In animal models of colitis, the administration of CpG ameliorated disease activity, probably mediated by the TLR9 receptor [12]. One can speculate in the context of colorectal cancer that the unwanted inflammatory activity is of microbial origin [82]. TLR antagonists in contrast are structural analogues of agonists which aim to dampen signalling. Currently, TLR4 antagonists are at clinical trial phase for the treatment of sepsis, with TLR7 and 9 antagonists under development in lupus [81]. TLR4 antagonists look to inhibit LPS recognition and are, therefore, a potentially suitable strategy for GI tract infections. However, to date, this has not been evaluated, and its usefulness is likely to vary for different Gram-negative stimuli. 

Alternative strategies towards microbially induced GI tract infections include altering the microbial component or targeting specific inflammatory pathways downstream of specific PRRs. With the understanding that gut homeostasis requires the interaction of an effective microbial/epithelial/immune circuit, with each component communicating with each other, therapeutic strategies are being developed which aim to restore defective aspects of the triad. Inhibition of effector T cells through the use of antibodies targeted against TNF-*α* is already proving effective in Crohn's disease [83]. Small molecular inhibitors of IKK have also been developed to specifically target the NF-*κ*B pathway. Other approaches include alteration of the GI bacterial cohort through antibiotic therapy or probiotics and genetically engineered microorganisms that inhibit immune or inflammatory responses.

## 6. Conclusion

Without doubt, understanding the interplay between host genetic factors, specifically pertaining to PRRs, and bacterial pathogenic mechanisms is crucial. This will be the cornerstone not only for a better understanding of bacterial pathogenesis, but also for drug development in the future. The importance of SNPs upon the outcome of these interactions is crucial to therapeutic developments and will play an important role in the development of personalized medicine.

## Figures and Tables

**Table 1:** Pattern recognition receptor activating ligands from *Salmonella spp. *and *Helicobacter pylori. *

PRR	*Salmonella spp. *ligand	*Helicobacter pylori *ligand
TLR1/2/6	Lipoproteins	Membrane protein HpaA
TLR4	Lipopolysaccharide (LPS)	Tetra-acylated LPS (poorly immunogenic) Hexa-acylated LPS
TLR5	Flagellin (FliC)	Flagellin (very poor stimulator)
TLR9	CpG rich repetitive elements in *Salmonella *DNA	Bacterial nucleic acid
NLRC4	Flagellin (FliC), T3SS protein PrgJ	?
NOD1	Peptidoglycan	Peptidoglycan

**Table 2:** TLR expression within the gastrointestinal tract. (Adapted from Fukata and Abreu 2008 and Gribar et al. 2008) [11, 19].

TLR	Stomach	Small intestine	Large intestine
1	Not detected	RNA	RNA
2	RNA	RNA/protein	RNA/protein
3	Not detected	RNA/protein	RNA/protein
4	RNA/protein	RNA/protein	RNA/protein
5	RNA/protein	RNA/protein	RNA/protein
6	Not detected	Not detected	RNA
7	Not detected	Not detected	RNA
8	Not detected	Not detected	RNA
9	RNA/protein	RNA/protein	RNA
10	Not detected	Not detected	Absent
11	Not detected	Not detected	Not detected
